# Examining Geographic Food Access, Food Insecurity, and Urbanicity among Diverse, Low-Income Participants in Austin, Texas

**DOI:** 10.3390/ijerph19095108

**Published:** 2022-04-22

**Authors:** Kathryn M. Janda, Nalini Ranjit, Deborah Salvo, Deanna M. Hoelscher, Aida Nielsen, Joy Casnovsky, Alexandra van den Berg

**Affiliations:** 1 UTHealth School of Public Health, Austin, TX 78701, USA; nalini.ranjit@uth.tmc.edu (N.R.); deanna.m.hoelscher@uth.tmc.edu (D.M.H.); aida.nielsen@uth.tmc.edu (A.N.); alexandra.e.vandenberg@uth.tmc.edu (A.v.d.B.); 2 Michael and Susan Dell Center for Healthy Living, Austin, TX 78701, USA; 3 Prevention Research Center, Brown School, Washington University in St. Louis, St. Louis, MO 63130, USA; dsalvo@wustl.edu; 4 Sustainable Food Center, Austin, TX 78702, USA; joy.casnovsky@gmail.com

**Keywords:** food insecurity, geographic food access, health disparities, urbanicity

## Abstract

The purpose of this study was to explore the association between geographic food access and food insecurity and the potential role of race/ethnicity, income, and urbanicity among a low-income, diverse sample in Central Texas. Utilizing a cross-sectional study design, secondary data analysis of an existing cohort was used to examine the association between food insecurity; geographic food access; and sociodemographic factors of race/ethnicity, income, urbanicity, and additional covariates using binomial logistic regression models. The existing cohort was recruited from lower-income communities in Travis County, Texas. The sample (N = 393) was predominantly Hispanic, lived in urban areas, and nearly 40% were food insecure. Geographic food access was not found to be significantly associated with food insecurity. However, rural residents had greater odds of being food insecure than urban residents. Also, participants who earned USD 45,000–64,999 and over USD 65,000 had lower odds of being food insecure than participants who earned under USD 25,000. These findings add to the inconsistent literature about the association between geographic food access and food insecurity and contribute to urbanicity and income disparities in food-insecurity literature. Future work should consider urbanicity, income, and utilize community-specific data to gain greater understanding of the association between geographic food access and food insecurity.

## 1. Introduction

### 1.1. Food Insecurity as a Public Health Issue

Food insecurity is a condition that occurs when individuals have uncertain or limited ability to acquire or lack availability of nutritious and safe foods in socially acceptable ways [1]. While the United States is a relatively high income and developed nation, food insecurity affected 11.1% of households in 2018 and 10.5% of households in 2019 [2,3]. The prevalence of food insecurity is higher in Texas, with 14% of Texan families identifying as food insecure from 2018 [3]. Austin/Travis County, Texas also had a higher prevalence of food insecurity than the national prevalence, with over 12.9% of families living in Travis County identifying as food insecure in 2018 and 12.8% in 2019 [4]. The high prevalence of food insecurity in Texas and the Austin area is alarming since being food insecure is associated with greater odds of having other health conditions, such as undernutrition, anemia, obesity and overweight, and chronic diseases [5,6,7,8,9]. Thus, food insecurity is a public health concern that needs to be further explored.

### 1.2. Geographic Food Access Conceptualization, and Disparities

Conceptually, food insecurity is comprised of four different components: availability, access, utilization, and stability over time [10]. Access consists of access to safe foods geographically and economically, and to culturally relevant foods [10]. One of the most commonly researched and discussed constructs of food insecurity is geographic food access [11]. Geographic food access refers to the ability of an individual to find food retail within their community [11]. Geographic food access has been seen as a critical and frequently measured and intervened upon construct when developing policies and interventions to reduce the prevalence of food insecurity within a community [11,12,13,14,15]. However, findings regarding the association between geographic food access and food insecurity have been inconsistent [16,17,18]. Some scholars have found that the association between geographic food access and food insecurity can vary based on community context and by types of measures of geographic food access [16,17,18].

One of the most notable disparities in the geographic food access literature is by race/ethnicity. In the systematic review done by Beaulac and colleagues (2009) analyzing peer-reviewed articles about limited geographic access to healthy foods from 1966 to 2007, areas with a predominant racial/ethnic minority population were found to be more likely to have limited geographic food access than predominantly non-Hispanic white communities [19]. The systematic review by Walker and colleagues (2010) built off the work by Beaulac (2009) and found that the racial/ethnic disparity was one of the most salient in the geographic food access literature [20]. Specifically, the authors identified that black neighborhoods were much less likely to have a supermarket than white neighborhoods in multiple urban areas including Philadelphia, New York, and Detroit [17,20,21,22,23]. However, black Americans are not the only racial/ethnic group that have been found to have limited geographic access to healthy foods. Hispanic communities also have been shown to have less geographic access to healthy foods than non-Hispanic whites [24]. In the work done by Lopez-Class and colleagues (2010), the authors found that Latinx communities in Erie County, New York had fewer supermarkets in their neighborhood, fewer public transportation opportunities, and had to pay more for fresh produce than non-Hispanic white communities in the area [24]. Grigsby-Toussaint and collaborators (2010) also identified that Latinx and other racial/ethnic minority communities in Chicago were less likely to have culturally relevant healthy foods available in their communities than predominantly white communities [25]. This work has been further corroborated by other scholars, noting that issues with food access disproportionately impact communities of color, and that this is the legacy of structural racism [26,27,28,29,30]. Thus, communities of color are more likely to have limited geographic access to food retailers and less likely to have culturally relevant healthy foods available in their communities than predominantly white communities.

Income disparities in geographic food access are also commonly discussed in the literature [19,20,31]. The aforementioned systematic review by Beaulac and scholars (2009) concluded that low-income areas have fewer supermarkets and large grocers compared to areas that were higher income [19]. Walker and colleagues (2010) found similar findings in their updated systematic review, citing that low-income communities were less likely to have supermarkets, but more likely to have small grocery stores that often had very limited healthy food offerings than higher income communities [20]. Sociologist Brian Thomas posited that the lack of supermarkets in low-income neighborhoods is due to a lack of purchasing power, that then prevents supermarket retailers from expanding into communities of need [32]. The well-established literature regarding income disparities and limited geographic food access contributed to the definition developed by the USDA of a food desert, which requires that the areas of limited geographic food access also are low income [33]. Additionally, many food access interventions have prioritized working in lower-income areas and communities in order to bridge this noted disparity [34,35,36,37,38].

Urban/rural disparities are also frequently discussed in the literature [39,40,41,42]. Specifically, areas with limited geographic food access are often seen in rural areas, however, they are often overlooked in the larger literature [40,41]. This is because rural areas are less densely populated and developed than urban areas, thus, there are fewer supermarkets and other large grocery stores [39,40,42]. Thus, there are different measures in quantifying areas with limited geographic food access for rural areas versus urban areas [40,41,43]. While there are numerous studies where the authors focus on rural communities [41], there needs to be more comparative work done that explores the role of limited geographic food access in rural and urban communities, such as the work done by Liese and colleagues (2014) and others that included both rural and urban areas in their food access analysis [14,19,20].

### 1.3. Gaps in the Literature

While there has been a wealth of published research on geographic food access, there are limitations to many of the utilized measures [43,44]. The majority of geographic food access research studies only utilize one measure of objective or perceived geographic food access and do not also incorporate demographic characteristics of the community being assessed [19,20]. This is problematic because solely using objective measures may not capture the intricacies of individual and community behaviors, and thus may be under- or overestimating areas with limited geographic food access [44,45].

Additionally, many of the published analyses only explore aggregated measures of geographic food access within an area, such as the number of supermarkets within a zip code, buffer, or census tract, rather than calculating individual distances, which would more accurately measure geographic food access [44,45]. Furthermore, in more recent studies that have incorporated a distance measure, they are often estimates of distances and utilized Euclidean measures rather than data directly from a community that utilized a network-distance based measure. [46] Thus, some scholars have called for more research to be conducted that incorporate both objective measures of geographic access and survey or qualitative data in order to more accurately and sensitively measure geographic food access within a community’s context [18,31,47,48].

Also, there needs to be more research done in contexts that contain areas across the urban–rural continuum in addition to various racial/ethnic and income groups [14]. Therefore, more research is needed that utilizes multiple measures of geographic food access and community context. This need has also been highlighted by Singleton and colleagues (2020), which called for incorporating an intersectionality approach, and simultaneously socioeconomic status, race/ethnicity, and geographic location [29]. To the best of our knowledge at the time of development of this paper, this is the first study to utilize community-specific survey data and objective measures of the food retail environment to examine the association between food insecurity and geographic food access while simultaneously taking into account race/ethnicity, income, and urbanicity.

### 1.4. Study Objective

The objective of this study was to explore the association between geographic food access and food insecurity and the potential role of individual-level measured indicators of race/ethnicity, income, and urbanicity, by utilizing a survey among a low-income racially/ethnically diverse sample in Central Texas and geographic data.

## 2. Materials and Methods

### 2.1. Parent Study Description, Study Design, and Sample

This study utilized a cross-sectional study design and data from the FRESH-Austin study [35]. The FRESH-Austin study description and baseline findings have been discussed elsewhere [35], but this paper presents secondary analyses utilizing the FRESH-Austin baseline survey. Participants were individuals who completed the baseline survey in October 2018–March 2019, were 18 years of age or older, were the primary food shoppers for their household, spoke English or Spanish, and lived in the greater Austin area (including areas outside of Travis County) [35]. The sample for this analysis only included cohort participants that lived in Travis County (*n* = 393) and had completed the baseline survey and provided a home address. Additionally, data regarding locations and types of food retail in Travis County were provided from the City of Austin Food Environment Analysis (FEA), which was a comprehensive program to map the food retail environment of Travis County, Texas. This study was approved by the UTHealth Institutional Review Board (HSC-SPH-18-0233) [35].

### 2.2. Variables of Interest

#### 2.2.1. Independent Variables: Geographic Food Access Measures

Geographic food access was measured by the presence of food retail near participants’ homes and distance from the participants’ homes to the closest food retail location by type. This was calculated by geocoding the home addresses of FRESH-Austin cohort participants, developing buffers around those home locations, and determining if food retail outlets are located in the buffers. Euclidean and network buffers (utilizing the Service Area Tool) at the 500 m, 1000 m, and 1500 m radii were calculated [49]. The “no trim” option was specified in line with the literature and given that multiple buffers were created [50]. The buffers were centered around each cohort participant’s home address, which were provided in the FRESH-Austin Baseline survey process. Additionally, distance from each participant’s home to the nearest large grocery stores/supermarkets and convenience stores was measured utilizing ArcGIS Closest Facility Tool for each type of food retail [49]. For the purpose of the logistic regression models, the presence of supermarkets/large grocery stores in 500 m and 1000 m were combined in order to have a sufficient sample size.

A food retail environment layer was developed for the analysis based on food retail location data from the City of Austin’s Food Environment Analysis of Travis County. Geographic food access was measured as a binary variable based on the presence of large grocery stores/supermarkets and convenience stores in the various size buffers. The majority of research investigating geographic food access looks at the presence of supermarkets/large grocery stores in an area [11,18,43,51]. Convenience stores were added to the analyses in order to have a slightly more nuanced view of the local food environment, and since convenience stores tend to offer more unhealthy food options [52,53]. Categories for food retail were defined based on the Baltimore Food Environment Report [54]. Large grocery stores/supermarkets were defined as over half of the items for sale were food items, and they offered additional services (e.g., pharmacy, optometrist, bill paying services, etc.). Convenience stores were defined as less than half of the items for sale were food items and typically no other additional services available.

#### 2.2.2. Dependent Variable: Food Insecurity Status

Food insecurity status was the dependent variable. Food insecurity status was measured based upon the two-item food insecurity screener in the baseline FRESH-Austin survey and dichotomized as food insecure or food secure. The two-item food insecurity screener was validated in a variety of low-income contexts and in clinical and non-clinical settings [55,56,57]. The two-item screener categorized households as food secure, sometimes food insecure, and often food insecure, but has been dichotomized in the literature as food secure and sometimes/often food insecure [55,56,57].

#### 2.2.3. Demographic Indicators: Race/Ethnicity, Income, and Urbanicity

A variety of demographic questions were also included in the FRESH-Austin Baseline survey and were included in the analysis. Race/ethnicity was self-reported in the baseline survey. Income was reported as a categorical variable, as under USD 25,000, between USD 25,000–44,999, USD 45,000–65,000, and over USD 65,000. Urbanicity was categorized based upon the census definition and US Department of Defense definition of urban areas based on population density [40,42,58]. Zip codes with a population density over 3000 people per square mile were categorized as urban, zip codes with a population density between 1000 and 3000 people per square mile were categorized as peri-urban, and zip codes with a population density of less than 1000 people per square mile were categorized as rural [58,59].

#### 2.2.4. Additional Covariates

Indicators such as employment, education status, and main mode of transportation were included in the analyses as potential covariates. Employment status, education, and main mode of transportation were all self-reported by participants in the FRESH-Austin baseline survey. Participants reported employment status as unemployed, part-time, full-time, or retired. Education status was reported by participants as less than high school, graduated high school/obtained GED, some college, and completed college or more. The main mode of transportation was reported as a personal car, ride share car or taxi, bicycle, walking, and public transit bus; however, this was then dichotomized into personal car or other mode of transportation for logistic regression analyses.

### 2.3. Overall Study Measures

The FRESH-Austin evaluation was designed to measure the impacts of the FFL program at various levels, these included measures at the individual, institutional, and community levels. A description of the measures is presented in Table 1. At the *individual level*, instruments included annual surveys with participants recruited into the cohort study, as well as wearable GPS and accelerometer device measurements among a subsample (*n* = 100) of those who were recruited to complete the survey. At the institutional or store level, this included audits of the stores as well as store counts of customers during a standardized period of time. At the community level, built environment audits were conducted.

### 2.4. Analysis Plan

Statistical analyses were performed utilizing Stata version 14 and ArcGIS [49,60]. Descriptive statistics included frequencies, and percentages were calculated for each categorical variable and indicator. Mean and standard deviations were calculated for each continuous variable. Dependent variables were assessed for normality using the Shapiro–Wilk test and visually. Categorical variables were checked for the potential need to collapse across categories. *t*-tests or Chi-square tests depending on the number of categories, or Mann–Whitney *U* tests (depending on normality) were used to compare differences across groups at α = 0.05 (*p* < 0.05). Additionally, logistic regression models were developed in order to examine the associations between geographic food access and food insecurity status among the FRESH-Austin cohort members. Tests of interaction between geographic food access and race/ethnicity and urbanicity were also conducted. This analysis plan was chosen in order to highlight the diversity of the sample and ensure that there was adequate distribution across variables. Multivariate logistic regression was selected given that this study explores the intersectional nature of demographic characteristics such as race/ethnicity, income, and urbanicity; we wanted to utilize an analytic approach that could include various variables in a single model (and test for potential interactions). Additionally, we wanted to make sure that the findings of our study were easily understandable by a broad audience, and logistic regression and descriptive statistics are commonly used methods in epidemiology and public health at large.

## 3. Results

### 3.1. Descriptives of the Sample

The final sample consisted of all participants that participated in the FRESH-Austin Baseline survey that lived in Travis County and provided their address (*n* = 393). Descriptive data are presented in Table 1. Participants in the FRESH-Austin Cohort reported were predominantly Hispanic (54.10%). Over 55% of participants lived in a zip code that was classified as urban. Almost 23% of participants reported an annual household income under USD 25,000, and 29% made over USD 65,000. Approximately 47% worked full-time, and 45% attained a college or advanced degree. Over 90% of participants reported using a personal car as their main form of transportation.

**Table 1:** Descriptive statistics for geographic food access, food insecurity, and various indicators and potential covariates among the FRESH-Austin Cohort.

Variable	*n* = 393	Frequency (%)	Mean (SD, Range)
**Demographic Indicators**			
Race/Ethnicity			
Black/African American	40	10.26	
Hispanic/Latino	211	54.10	
White	126	32.31	
Other	13	3.33	
Urbanicity			
Urban (>3000 people/mile^2^)	216	55.38	
Peri-Urban (1000–3000 people/mile^2^)	92	23.59	
Rural (<1000 people/mile^2^)	82	21.03	
Household Income			
Under USD 25,000	86	22.93	
USD 25,000–44,999	109	29.07	
USD 45,000–64,999	69	18.40	
USD 65,000+	111	29.60	
**Additional Potential Covariates**			
Employment Status			
Unemployed	102	26.02	
Part-time	67	17.09	
Full-time	185	47.19	
Retired	38	9.69	
Education			
Less than High School	48	12.31	
Completed High School	82	21.03	
Some College/Tech Training	83	21.28	
Completed College or Advanced Degree	177	45.38	
Main Mode of Transportation			
Personal Car	358	91.09	
Ride Share/Taxi	4	1.01	
Walking	7	1.78	
Biking	5	1.27	
Public Transportation	16	4.07	
**Geographic Food Access Variables**			
Presence of Large Grocery Store/Supermarket			
Access within 500 m Network Buffer	3	0.76	
Access within 1000 m Network Buffer Only	48	12.21	
Access within 1500 m Network Buffer Only	51	12.98	
No access within any of the Network Buffers	300	76.34	
Presence of Convenience Stores			
Access within 500 m Network Buffer	107	27.23	
Access within 1000 m Network Buffer Only	147	37.4	
Access within 1500 m Network Buffer Only	62	15.78	
No access within any of the network buffers	77	19.59	
Average Distance to Food Retail in Miles			
Average Distance to Closest Supermarket/Large Grocery Store			1.66 (1.30, 0.24–15.83)
Average Distance to Closest Convenience Store			0.67 (0.61, 0.00–5.67)
**Food Insecurity**			
Never Food Insecure	241	60.40	
Sometimes or Often Food Insecure	158	39.60	

Only three participants had a supermarket/large grocery store located in their smallest (500 m) network buffer. The vast majority of participants (76.49%) did not have a supermarket located in their 500 m, 1000 m, or 1500 m network buffers. However, over 80% of participants had a convenience store located within 1500 m of their home. These differences in proximity are also evident in the average distance to closest supermarket and corner stores, with participants living an average of 1.66 miles away from the closest supermarket/large grocery store, and 0.67 miles away from the closest convenience store. Nearly 40% of participants reported being sometimes or often food insecure.

### 3.2. Results from Logistic Regression Models

The findings for the logistic regression models are presented in Table 2. Model I depicts unadjusted associations for the geographic food access variables of presence of specific food retail within buffers, average distance to the nearest supermarket or convenience store, race/ethnicity, and urbanicity variables. Model II includes the variables presented in Model I and additionally includes the potential covariates of income, employment status, education, and main mode of transportation. Tests of interactions between geographic food access and race/ethnicity and urbanicity variables were conducted, but there were no statistically significant interactions.

Findings from the unadjusted model found that geographic food access was not statistically significant with food insecurity among cohort participants within this relatively small geographic area. However, participants who identified as Hispanic had 2.79 times greater odds (*p* < 0.01) of being food insecure than cohort participants who identified non-Hispanic white. In the adjusted model, residents of rural areas had 2.07 times greater odds (*p* < 0.05) of being food insecure than cohort participants from urban areas. Income was a significant covariate, with participants who earned between USD 45,000–64,999 (OR = 0.25, *p* < 0.01) and over USD 65,000 (OR = 0.09, *p* < 0.01) had lower odds of being food insecure than cohort participants who had an annual income under USD 25,000.

## 4. Discussion

### 4.1. Summary of Overall Findings

The results of the descriptive statistics and logistic regression models indicated that there are various factors that were associated with food insecurity among the FRESH-Austin Cohort. Of note, the prevalence of food insecurity in the FRESH-Austin Cohort (39.60%) is more than double the prevalence of food insecurity for Austin/Travis County in 2018 (12.9%) [4]. Geographic food access was not found to be associated with food insecurity at a statistically significant level in the adjusted or unadjusted models, which adds to inconsistent findings found in the established literature [16,17,18]. Ma and colleagues (2016) posited that perhaps perceived geographic food access could be a better indicator than objective measures of geographic food access and should be explored in future research [18].

Survey participants who identified as Hispanic had greater odds (OR = 2.79, *p* < 0.01) of being food insecure than their non-Hispanic white counterparts in the unadjusted model, but the association was no longer significant once adjusting for income, employment, education, and main mode of transportation, contrary to the literature base [19,20,23,31,39,61]. This lack of association could be due to the composition of the sample, which was predominantly non-Hispanic white. Future analysis is needed in order to draw more conclusive findings. Participants who lived in rural zip codes had greater odds of being food insecure than those who lived in urban zip codes in both the unadjusted and adjusted models. This finding is novel, since the majority of food insecurity and geographic food access research is focused on either rural or urban populations, rather than a cohort that contains multiple urbanicity classifications [39,40,42,62,63]. Income had statistically significant associations with food insecurity, which is consistent with the established literature [19,20,31].

### 4.2. Strengths and Limitations

#### 4.2.1. Strengths

This study has numerous strengths and potential contributions to the literature. Previous studies typically only utilized census data with food retail or aggregated data to measure objective geographic food access and did not use additional data sources to provide greater context [19,20,45,64]. This lack of community-driven and individual-level data has been identified by researchers, who have often stated the need for utilizing multiple sources of data [14,31,47,65,66].

As previously mentioned, the majority of food insecurity and geographic food access research is focused solely on an urban or rural area. Thus, the majority of research does not compare geographic food access and food insecurity across multiple urbanicity classifications (urban, peri-urban, and rural), as was done in this analysis [39,40,42,62,63]. Therefore, this study provides a valuable contribution to the literature base.

#### 4.2.2. Limitations

However, there are notable limitations in this study. For instance, while this study utilized individual level data for location of participants, proximity to food retail, and demographic factors and indicators, it did not incorporate confirmed shopping behaviors. Additionally, this study utilized purposeful sampling with customers of specific non-traditional food retail outlets, in areas that were geographically proximal to those outlets, and areas that were similar demographically to those that were proximal to the outlets. Thus, the resulting sample predominantly resided in the Eastern Crescent of Austin (which is mainly low-income), were majority Hispanic, and had a much higher prevalence of food insecurity than other city data; therefore, these findings may not be generalizable to other communities or other areas in the greater Austin area [4]. However, even when analyses were restricted to the geographically exposed and control groups of the sample, the same significant findings were found with the same directionality and similar magnitude. Future research could explore these associations with a sample that includes higher-income areas of Austin in order to have a more representative sample.

Additionally, this study utilized pre-COVID-19 pandemic data. Therefore, this study sheds light onto phenomena that could be relevant to COVID-19 and post-COVID-19 era findings; however, future research should seek to explore these associations in this new climate. Yet, despite these limitations, this study fills notable gaps in the literature by utilizing multiple data sources and including demographic indicators and covariates, and accounting for urbanicity in the analysis.

## 5. Conclusions

Given that urbanicity and income were statistically significantly associated with food insecurity, future research regarding the association between geographic food access and food insecurity should be mindful of urbanicity, income, and the potential role of perceived geographic food access. Urbanicity should be taken into account when exploring the association between geographic food access and food insecurity in areas that are not strictly urban or rural. By including urbanicity, future research could have a more nuanced understanding of how the association between geographic food access and food insecurity exist in different environments. Also, the inconsistent literature regarding geographic food access and food insecurity suggest that other measures of food access should be considered, such as perceived geographic food access or economic food access. These measures could be particularly relevant in the age of COVID-19, which limited geographic food access through various stay-at-home orders and also had greater prevalence of food insecurity. Thus, further research is needed in order to gain a more consistent literature base and greater insight as to how geographic food access and food insecurity are related in various settings.

## Figures and Tables

**Table 2:** Logistic regression examining the association between geographic food access, food insecurity status, and various indicators and potential covariates.

*n* = 393	Model I	Model II
Variable (reference category for categorical variables)	Unadjusted OR (CI)	Adjusted OR (CI)
**Variables Introduced in Model I**		
Presence of Supermarket/Large Grocery Store (Referent = 500 m or 1000 m Network Buffer)		
Presence within 1500 m Buffer	0.97 (0.38–2.48)	1.33 (0.44–4.01)
Not Present in Any Buffer	1.01 (0.45–2.31)	1.03 (0.42–2.57)
Presence of Convenience Store (Referent = 500 m Network Buffer)		
Presence within 1000 m Buffer	0.70 (0.39–1.24)	0.85 (0.43–1.67)
Presence within 1500 m Buffer	1.24 (0.56–2.77)	1.15 (0.46–2.90)
Not Present in Any Buffer	1.03 (0.38–2.82)	1.12 (0.36–3.46)
Distance to Food Retail in Miles		
Distance to Closest Supermarket/Large Grocery Store	0.94 (0.74–1.20)	0.95 (0.75–1.21)
Distance to Closest Convenience Store	0.93 (0.49–1.75)	1.04 (0.52–2.07)
Race/Ethnicity (Referent = White)		
Black/African American	1.45 (0.65–3.24)	0.91 (0.34–2.40)
Hispanic or Latino	2.79 (1.67–4.67) **	1.05 (0.52–2.13)
Other	1.38 (0.39–4.92)	1.13 (0.26–4.97)
Urban/Rural Status (Referent = Urban)		
Peri-Urban(Between 1000–3000 people/square mile)	0.97 (0.56–1.68)	0.89 (0.47–1.67)
Rural (<1000 people/square mile)	1.70 (0.96–3.03)	2.07 (1.04–4.09) *
**Variables Introduced in Model II**		
Household Income (Referent = Under $25,000)		
USD 25,000–44,999		0.64 (0.34–1.23)
USD 45,000–64,999		0.25 (0.11–0.58) **
Over USD 65,000		0.09 (0.03–0.22) **
Employment Status (Referent = Unemployed)		
Part-Time		0.82 (0.39–1.74)
Full-Time		0.67 (0.35–1.27)
Retired		0.61 (0.22–1.67)
Education (Referent = Less Than High School)		
Completed High School or GED		0.68 (0.29–1.60)
Some College		1.24 (0.48–3.21)
College or Advanced Degree		0.62 (0.24–1.59)
Main Mode of Transportation (Referent = Personal car)		
Other Form of Transport		2.19 (0.94–5.11)

* p < 0.05, ** *p* < 0.01.

## Data Availability

Data can be shared by request and contacting the authors.
